# BLSAM-TIP: Improved and robust identification of tyrosinase inhibitory peptides by integrating bidirectional LSTM with self-attention mechanism

**DOI:** 10.1371/journal.pone.0333614

**Published:** 2025-10-08

**Authors:** Saeed Ahmed, Nalini Schaduangrat, Pramote Chumnanpuen, S. M. Hasan Mahmud, Kah Ong Michael Goh, Watshara Shoombuatong

**Affiliations:** 1 Center for Research Innovation and Biomedical Informatics, Faculty of Medical Technology, Mahidol University, Bangkok, Thailand; 2 Department of Computer Science, University of Swabi, Swabi, Pakistan; 3 Department of Zoology, Faculty of Science, Kasetsart University, Bangkok, Thailand; 4 Kasetsart University International College (KUIC), Kasetsart University, Bangkok, Thailand; 5 Department of Software Engineering, Daffodil International University, Daffodil Smart City (DSC), Birulia, Savar, Dhaka, Bangladesh; 6 Faculty of Information Science and Technology, Multimedia University, Jalan Ayer Keroh Lama, Melaka, Malaysia; Hebei University of Technology, CHINA

## Abstract

Tyrosinase plays a central role in melanin biosynthesis, and its dysregulation has been implicated in the pathogenesis of various pigmentation disorders. The precise identification of tyrosinase inhibitory peptides (TIPs) is critical, as these bioactive molecules hold significant potential for therapeutic and cosmetic applications, including the treatment of hyperpigmentation and the development of skin-whitening agents. To date, computational methods have received significant attention as a complement to experimental methods for the *in silico* identification of TIPs, reducing the need for extensive material resources and labor-intensive processes. In this study, we propose an innovative computational approach, BLSAM-TIP, which combines a bidirectional long short-term memory (BiLSTM) network and a self-attention mechanism (SAM) for accurate and large-scale identification of TIPs. In BLSAM-TIP, we first employed various multi-source feature embeddings, including conventional feature encodings, natural language processing-based encodings, and protein language model-based encodings, to encode comprehensive information about TIPs. Secondly, we integrated these feature embeddings to enhance feature representation, while a feature selection method was applied to optimize the hybrid features. Thirdly, the BiLSTM-SAM architecture was specially developed to highlight the crucial features. Finally, the features from BiLSTM-SAM was fed to deep neural networks (DNN) in order to identify TIPs. Experimental results on an independent test dataset demonstrate that BLSAM-TIP attains superior predictive performance compared to existing methods, with a balanced accuracy of 0.936, MCC of 0.922, and AUC of 0.988. These results indicate that this new method is an accurate and efficient tool for identifying TIPs. Our proposed method is available at https://github.com/saeed344/BLSAM-TIP for TIP identification and reproducibility purposes.

## 1. Introduction

Tyrosinase is a crucial enzyme involved in the biosynthesis of melanin, catalyzing the initial steps of melanogenesis in mammals and contributing to enzymatic browning in fruits and vegetables [1–3]. This copper-containing oxidase facilitates the oxidation of phenolic compounds, leading to the formation of melanin, which plays an essential role in pigmentation and protection against UV radiation [1]. However, excessive melanin production can result in hyperpigmentation disorders, and the browning of fruits during storage can lead to economic losses in the food industry. Consequently, there is a significant interest in developing tyrosinase inhibitors as therapeutic agents for skin conditions and as preservatives in food products [4,5]. The overproduction of melanin can lead to hyperpigmentation disorders, such as melasma and age spots, which pose both cosmetic and medical concerns [6,7]. As a result, there has been growing interest in identifying effective inhibitors of tyrosinase to mitigate these conditions [8,9]. The search for effective tyrosinase inhibitors has led to the identification of various natural and synthetic compounds. These inhibitors can be classified into different categories based on their mechanisms of action and chemical structures. Some inhibitors act as competitive or non-competitive agents, while others may irreversibly bind to the enzyme, effectively inactivating it during catalysis. For instance, kojic acid is one of the most well-studied tyrosinase inhibitors and serves as a benchmark for evaluating the efficacy of new compounds [10,11].

Tyrosinase inhibitory peptides (TIPs) have emerged as promising candidates for reducing melanin production. These peptides, typically composed of 3–20 amino acids, can effectively inhibit tyrosinase activity. Recent studies have demonstrated that various bioactive peptides derived from natural sources exhibit strong tyrosinase inhibitory properties, offering a safer alternative to traditional chemical inhibitors such as hydroquinone and kojic acid, which may cause adverse side effects [12,13]. The mechanisms by which TIPs exert their inhibitory effects are multifaceted. These peptides can bind to the active site of tyrosinase, leading to competitive inhibition or, in some cases, irreversible inhibition. Additionally, TIPs may modulate signaling pathways involved in melanogenesis, further enhancing their effectiveness in treating hyperpigmentation [9,14].

The methodologies employed for identifying TIPs can be broadly categorized into *in vitro* and *in silico* approaches, each with distinct advantages and limitations. Current experimental methods face significant challenges in high-throughput screening due to their labor-intensive and expensive nature [8,15]. Recently, advancements in computational methods, such as machine learning (ML) algorithms, have facilitated the prediction and identification of novel TIPs. These approaches allow researchers to screen thousands of peptides based on their structural properties and predicted anti-tyrosinase activities, demonstrating high accuracy rates [8,12,15]. By integrating bioinformatics with peptide research, these advancements are driving more precise and efficient strategies for tyrosinase inhibition. Notable examples include ML-based methods such as TIP-KNN and TIP-RF [15], as well as TIPred [8]. Comprehensive details on these cutting-edge techniques are provided in earlier studies [8]. Despite ongoing improvements in the predictive performance of these advanced methods [8,15], their practical effectiveness in real-world applications remains inadequate. Key challenges include the limited availability of known TIPs and issues related to imbalanced learning.

Although the existing methods facilitate the identification of TIPs, several challenges remain to be addressed. First, relying on single feature descriptor is insufficient for capturing the comprehensive information of TIPs [16–20]. Second, protein language models (PLMs), inspired by natural language models (LMs), have recently shown effectiveness in generating peptide sequence representations [19,21,22]. Since PLMs are pre-trained on extensive protein databases such as BFD [23,24], UniRef [25], and Pfam [26], which collectively contain over a billion protein sequences, they can extract comprehensive and valuable information. Regrettably, no studies have yet employed PLMs to generate feature representations for TIPs. Third, the imbalance between TIPs and non-TIPs in datasets can adversely affect the prediction performance of the models. Finally, the overall prediction accuracy and robustness of existing methods remains inadequate, highlighting the need for further improvements.

**Fig 1:**
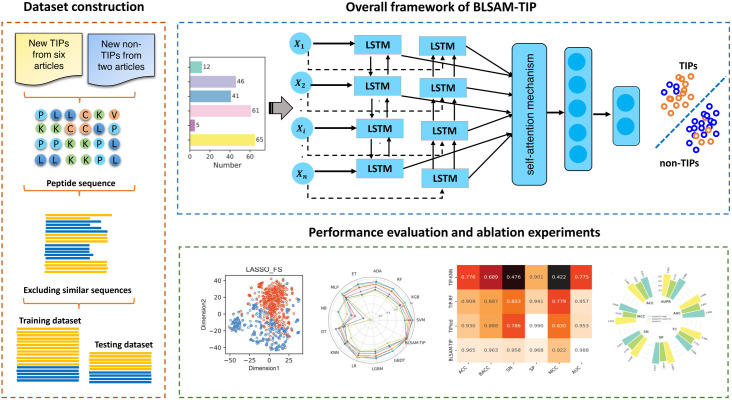
An overview of BLSAM-TIP for identifying TIPs. **(A)** Data construction. **(B)** Overall framework of BLSAM-TIP. **(C)** Performance evaluation and ablation experiments.

To address these deficiencies, a novel computational approach, termed BLSAM-TIP, leveraging a combination of bidirectional long short-term memory (BiLSTM) and a self-attention mechanism (SAM), is proposed for the accurate and large-scale identification of TIPs (**Fig 1**). The major contributions of the proposed model can be summarized in the following four aspects. First, to capture multi-view and comprehensive information about TIPs, various feature encoding schemes were employed, encompassing sequential information, graphical information, statistical information, contextual information, and protein semantic information. Second, the synthetic minority oversampling technique (SMOTE) was utilized to address the impact of data imbalance on the model’s performance. Additionally, the least absolute shrinkage and selection operator (LASSO) method was applied to optimize the combined features, potentially enhancing the model performance. Third, the BiLSTM-SAM-DNN architecture was specially constructed to reduce interference from irrelevant information and subsequently employed to identify TIPs. Fourth, benchmark experiments on the independent test set illustrated that BLSAM-TIP significantly outperformed existing state-of-the-art methods, achieving a balanced accuracy (BACC) of 0.936, Matthew’s correlation coefficient (MCC) of 0.870, and an area under the receiver-operating curve (AUC) of 0.988.

## 2. Materials and methods

### 2.1 Data collection and curation

The existing predictors for TIPs were developed and fine-tuned using a limited dataset of TIPs and non-TIPs, as detailed in S1 Table. Developing a high-accuracy predictive model necessitates a larger sample size [27–29]. To construct an updated and high-quality dataset, specific filtering criteria were applied to the initial TIPs and non-TIPs: (i) peptide sequences containing unusual letters such as ‘B’, ‘U’, ‘X’, or ‘Z’ were eliminated; and (ii) duplicate peptide sequences were removed. After applying these filters, a refined dataset comprising 206 TIPs and 502 non-TIPs was compiled. These sequences were sourced from our previous research [8] and six recently published studies [8,12,15,30–34], while 401 non-TIPs were taken from our earlier work [8] and Qin et al. [35]. Since TIPs are typically shorter than 20 amino acid residues [9], both TIP and non-TIP datasets were restricted to peptide lengths ranging from 2 to 20 amino acids. For the establishment of training and independent test datasets, we adhered to the criteria set forth by Charoenkwan et al. [8]. The training dataset consisted of 164 TIPs and 401 non-TIPs, while the independent test dataset included 42 TIPs and 101 non-TIPs. Additional details regarding the composition of the training and independent test datasets can be found in S1 Table.

### 2.2 Feature encoding scheme

To capture comprehensive information about TIPs, we employed six feature encoding methods from different perspectives, including conventional feature encodings, NLP-based encodings, and protein language model-based encodings. For conventional feature encoding, we applied a novel feature extraction method called FEGS, which is capable of capturing graphical and statistical information [36]. FEGS integrates two interpretable feature descriptors (i.e., amino acid composition (AAC) and dipeptide composition (DPC)) with the physicochemical properties of amino acids. Based on FEGS, any peptide sequence is encoded as a 578-D feature vector. In the recent years, embedding methods inspired by NLP techniques have gained attention in the field of bioinformatics and computational biology [37–40]. These methods provide contextual information for peptide sequences [37,38,41]. Among them, FastText is a powerful embedding method that leverages morphological information to address the issue of out-of-vocabulary words [42,43], thereby improving performance in downstream tasks. Herein, FastText generates a 120-D feature vector for peptide sequences. With advancements in NLP techniques and the availability of millions of protein sequences, PLMs have been increasingly employed as embedding extractors. In this study, we utilized four well-known PLMs, including bidirectional encoder representations from transformers (BERT) [44], ProtT5-U50, ProtT5-BFD, and ESM-2, to encode peptide sequences into feature embeddings (i.e., distributed vector representations). The text-to-text transfer transformer (T5) [45] architecture was used to develop both ProtT5-BFD and ProtT5-UR50. ProtT5-UR50 was trained on Uniref50 [25], which contains 45 million protein sequences, while ProtT5-BFD was trained on BFD [46], a database comprising 2.1 billion protein sequences. to account for the relatively small size of the training dataset, we used ESM-2, which was trained on the UR50/D 2021_04 dataset (called esm2_t6_8M_UR50D). Additionally, we used the esm2_t33_650M_UR50D model [47], which is based on the BERT architecture and was trained on Uniref50. Using these PLMs, peptide sequences were encoded as feature vectors of varying dimensions: 768-D, 1024-D, 1024-D, and 320-D for BERT, ProtT5-U50, ProtT5-BFD, and ESM-2, respectively.

### 2.3 Feature selection method

In the field of bioinformatics, feature selection plays an important role in enhancing model efficiency and addressing overfitting [48–50]. Robert Tibshirani introduced a well-regarded feature selection method called LASSO, which can perform both feature selection and regularization. This method has been effective in identifying beneficial features from high-dimensional data [51]. Given X and y as the features and classes, the linear regression model is defined as follows:


y= βX+ε (1)

where $\text{X}=\;{\text{X}}_{\text{1}},\;{\text{X}}_{\text{2}},\;\ldots ,{\text{X}}_{p},\;$ $\text{y}=\;{{(\text{y}}_{\text{1}},\;{\text{y}}_{\text{2}},\;\ldots ,{\text{y}}_{p})}^{T}$, $\beta =\;{{(\beta }_{\text{1}},\;\beta_{\text{2}},\;\ldots ,\beta_{p})}^{T}$, and ε is the error term. In the LASSO method, the goal is to determine the optimal value of β with a special penalty constraint. The LASSO estimation is defined as follows:


$$L_{\lambda }(\beta )={\left\|y-\;\beta \text{X}\right\|}_{\text{2}}^{\text{2}}+{\lambda \left\|\beta \right\|}_{\text{1}}$$ (2)

$$\begin{array}{c} {\left\|\beta \right\|}_{\text{1}}=\sum {\mathrm{\backslash nolimits}}_{k=\text{1}}^{p}\left|\beta_{k}\right| \end{array}$$ (3)

where ${\left\|\cdot \right\|}_{\text{2}}$ represents the Euclidean norm.

### 2.4 Bidirectional long short-term memory and self-attention mechanism

Long short-term memory network (LSTM) can learn long-term sequential features without requiring a large number of features, unlike traditional ML models, that often depend on additional features to improve model performance. The LSTM method was developed to address the vanishing gradient problem [52,53], a challenge encountered in recurrent neural networks (RNNs) [54]. LSTMs use memory cells to decide which information to retain and which to discard, enabling them to capture long-range contextual information effectively. The structure of an LSTM typically contains three main components, such as the forget gate ($f_{t}$), the input gate ($i_{t}$), and the memory cell ($C_{t}$). At time t, the formulations of the LSTM structure can be defined as follows:


$$i_{t}=\;\sigma (W_{i}[h_{t-\text{1}},\;F_{t}]+b_{i})$$ (4)

$$f_{t}=\;\sigma (W_{f}[h_{t-\text{1}},\;F_{t}]+b_{f})$$ (5)

$$\tilde{C_{t}}=\;tanh(W_{C}[h_{t-\text{1}},\;F_{t}]+b_{C})$$ (6)

$$\begin{array}{c} C_{t}=F_{t}⨂C_{t-\text{1}}+i_{t}⊗\tilde{C_{t}} \end{array}$$ (7)

$$o_{t}=\;\sigma (W_{o}[h_{t-\text{1}},\;F_{t}]+b_{o})$$ (8)

$$h_{t}=\;o_{t}⨂\text{tanh}(C_{t})$$ (9)

$$\sigma =\;\frac{\text{1}}{\text{1}+e^{-z}}$$ (10)

where $W_{i}$, $W_{f}$, and $W_{o}$ represent the weights of $i_{t}$, $f_{t}$, and $o_{t}$, respectively, while $b_{i}$, $b_{f}$, and $b_{o}$ are the biases of input gate, forget gate, and output gate, respectively. $C_{t}$ is the updated cell state, generated based on the previous cell state. . Rather than using LSTM, we applied BiLSTM, which consists of two LSTM layers – one processing sequences in the forward direction and the other in the backward direction. This design enables BiLTSM to capture both future/upcoming and historical contexts, allowing it to extract not only past information but also future features, thereby achieving better prediction performance compared to standard LSTMs.

### 2.5 Self-attention mechanism

To date, the attention mechanism has effectively helped models highlight important parts of sentences in several NLP tasks, such as aspect-level sentiment classification. Specifically, the attention mechanism can automatically extract significant word embeddings from text sequences during model training [55,56]. Several previous studies have demonstrated its successful application in enhancing model performance in bioinformatics and computational biology [48,57,58]. Thus, after obtaining the features generated from BiLSTM, we employed the SAM to strengthen specific BiLSTM-based feature representations. In the SAM structure, given an input, it can generate three standard matrices Query (Q), Key (K), and Value (V), which is calculated as follows:


$$Attention(Q,K,V)=softmax(\frac{QK^{T}}{\sqrt{d}})$$ (11)

where $Q=W_{q}X$, $K=W_{k}X$, and $V=W_{v}X$. Here, d represents the dimensionality of Q and K, while $W_{q}$, $W_{k}$, and $W_{v}$ are the weight matrices used to compute Q, K, and V, respectively.

### 2.6 The overall framework and performance of BLSAM-TIP

**Fig 1** illustrates the overall framework and performance of the proposed BLSAM-TIP model for identifying TIPs. As shown in **Fig 1**, BLSAM-TIP is a DL-based prediction model where the input is a query peptide sequence, and the output is the confidence score for TIP identification. The BLSAM-TIP framework consists of two main procedures: (i) multi-view feature extraction and optimization, and (ii) TIP identification using the BiLSTM-SAM-DNN architecture.

#### Procedure I: Multi-view feature extraction and optimization.

The input peptide sequence is processed using various feature encoding methods, including FastText, BERT, ProtT5-U50, ProtT5-BFD, ESM-2, and FEGS. These methods generate feature vectors of dimensions 120-D, 768-D, 1024-D, 1024-D, 320-D, and 578-D, respectively. These diverse feature representations capture different types of information, such as sequential information, graphical information, contextual information, and protein semantic information. To comprehensively represent multi-view information beneficial for TIP identification, we combined the above-mentioned feature representations. Given the imbalance between TIP and non-TIP samples (i.e., 158 TIPs and 408 non-TIPs), the learning accuracy and model performance might be imparied [59,60]. Thus, to address data imbalance, the SMOTE method was employed to oversample TIPs [61]. As a result, we obtained a hybrid feature vector containing 3848 features. To eliminate noise and irrelevant information, several well-known feature selection methods were applied, generating various feature subsets. Finally, the optimal feature subset was selected based on the best-performing cross-validation MCC value.

#### Procedure II: TIP identification using the BiLSTM-SAM-DNN architecture.

The BiLSTM-SAM architecture, which combines BiLSTM and SAM, was specifically designed to mitigate interference from irrelevant information and enhance prediction performance. Finally, the BiLSTM-SAM-based feature representations were input into deep neural networks (DNN) for the identification of TIPs [48,62]. The performance of BLSAM-TIP and related prediction models was evaluated using several metrics, including BACC, AUC, MCC, F1, area under the precision-recall curve (AUPR), sensitivity (SN), and specificity (SP) [63–68]. Additional details about these seven performance metrics are provided in the Supplementary information.

## 3. Results and discussion

### 3.1 Performance evaluation of different feature representations

Here, we selected six feature extraction methods (i.e., FastText, BERT, ProtT5-U50, ProtT5-BFD, ESM-2, and FEGS) to capture critical information about TIPs from multiple perspectives, including sequential, graphical, semantic, and evolutionary information. Specifically, the feature representations derived from these methods were processed using the SMOTE method to address the class-imbalance problem in the training dataset (164 TIPs and 401 non-TIPs) [59,60]. Finally, the processed feature representations were fed into the BiLSTM-SAM architecture. To evaluate the representational capability of these feature extraction methods, we evaluated their performance in terms of ACC, AUC, AUPR, SN, SP, MCC, and F1 scores through a five-fold cross-validation test, as detailed in Table 1. From Table 1, the MCC values of FastText, BERT, ProtT5-U50, ProtT5-BFD, ESM-2, and FEGS are 0.904, 0.963, 0.981, 0.964, 0.968, and 0.743, respectively. Interestingly, the top-three feature representations were obtained from PLMs. Among these, ProtT5-U50 provided the best feature representation, achieving ACC, SN, SP, F1, AUC, and AUPR values of 0.990, 0.985, 0.995, 0.990, 0.998, and 0.998, respectively.

**Table 1:** Performance comparison of different feature representations over the training dataset.

Feature	ACC	SN	SP	MCC	F1	AUC	AUPR
FastText	0.951	0.968	0.934	0.904	0.952	0.988	0.985
BERT	0.982	0.983	0.980	0.963	0.982	0.991	0.991
ProtT5-U50	0.990	0.985	0.995	0.981	0.990	0.998	0.998
ProtT5-BFD	0.982	0.985	0.978	0.964	0.982	0.996	0.996
ESM-2	0.984	0.985	0.983	0.968	0.984	0.996	0.997
FEGS	0.854	0.995	0.714	0.743	0.879	0.968	0.963
Hybrid	0.947	0.990	0.905	0.900	0.951	0.991	0.991

### 3.2 Determination of optimal feature subsets

Rather than employing only the best-performing feature (ProtT5-U50) to develop the final model, we combined six feature representations into a single hybrid feature vector (named Hybrid) to improve the feature space and capture more comprehensive information about TIPs. However, the performance of the Hybrid was lower than that of ProtT5-U50 (as shown in Table 1). The possible reason for this decline is that using the Hybrid significantly increased data dimensionality, introducing noise and potentially degrading the model performance. To address this challenge, we applied eight distinct feature selection methods [50,69–71] to the training dataset. These methods included LASSO, mRMR, random projection (RP), truncated singular value decomposition (TSVD), elastic net (EN), graph autoencoders (GAE), principal component analysis (PCA), spectral embedding (SE), which generated eight different feature subsets. For ease of discussion, these feature subsets are referred to as LASSO_FS, mRMR_FS, RP_FS, TSVD_FS, EN_FS, GAE_FS, PCA_FS, and SE_FS, respectively. Specifically, we trained nine individual BiLSTM-SAM-based models, each using one of the feature subsets. The prediction results were evaluated over the cross-validation and independent test on the training and independent test datasets, respectively. The feature dimensions of the subsets were 259, 400, 230, 700, 700, 301, 37, and 500 for EN_FS, GAE_FS, LASSO_FS, mRMR_FS, PCA_FS, RP_FS, SE_FS, and TSVD_FS, respectively. The optimal feature subset, determined by the highest cross-validation MCC, was considered the most effective for TIP identification.

**Fig 2:**
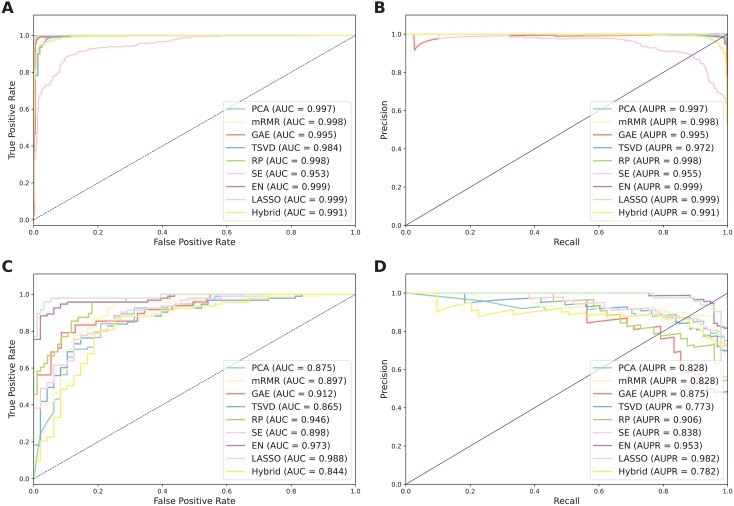
Performance comparison of different feature selection methods. Comparisons of the ROC curve, AUC value, PR curve, and AUPR value on the training (A, B) and independent test (C, D) datasets.

Fig 2 and Table 2 summarize the prediction results of the selected feature selection methods and the control, where the control refers to the hybrid feature vector. The performance of all the feature subsets exceeded that of the control, with the sole exception of the SE-based feature subset. This indicates the effectiveness of the feature selection methods. As seen in Table 2, five feature selection methods, encompassing EN_FS, LASSO_FS, RP_FS, PCA_FS, and mRMR_FS, achieved cross-validation MCC values greater than 0.980. To evaluate its generalization ability, its performance was further assessed on the independent test dataset. The corresponding MCC values for these methods were 0.841, 0.922, 0.697, 0.651, and 0.712, respectively. Overall, LASSO_FS exhibited optimal performance across both validation strategies. Notably, the MCC values of LASSO_FS were 9.02% and 33.44% higher than the control in terms of the cross-validation and independent tests, respectively. Moreover, on the independent test, the ACC, SN, SP, F1, AUC, AUPR values of LASSO_FS were 16.20, 14.58, 17.02, 33.44, 21.26, 14.32, and 20.07%, respectively, higher than the control. Thus, we utilized the LASSO_FS subset, comprising 230 selected features, to optimize the proposed BLSAM-TIP model herein.

**Table 2:** Performance of different feature selection methods over the cross-validation and independent tests.

Evaluation strategy	Method	ACC	SN	SP	MCC	F1	AUC	AUPR
Cross-validation	PCA	0.991	0.985	0.998	0.984	0.991	0.997	0.997
	mRMR	0.991	0.988	0.995	0.983	0.991	0.998	0.998
	GAE	0.983	0.988	0.978	0.966	0.983	0.995	0.995
	TSVD	0.968	0.962	0.970	0.927	0.949	0.984	0.972
	RP	0.994	0.995	0.993	0.988	0.994	0.998	0.998
	SE	0.888	0.904	0.872	0.782	0.890	0.953	0.955
	EN	0.998	1.000	0.995	0.995	0.998	0.999	0.999
	LASSO	0.995	0.995	0.995	0.990	0.995	0.999	0.999
	Control	0.947	0.990	0.905	0.900	0.951	0.991	0.991
Independent test	PCA	0.845	0.750	0.894	0.651	0.766	0.875	0.828
	mRMR	0.873	0.771	0.926	0.712	0.804	0.897	0.828
	GAE	0.852	0.792	0.883	0.671	0.784	0.912	0.875
	TSVD	0.803	0.687	0.862	0.555	0.702	0.865	0.773
	RP	0.866	0.771	0.915	0.697	0.796	0.946	0.906
	SE	0.817	0.750	0.851	0.595	0.735	0.898	0.838
	EN	0.930	0.875	0.957	0.841	0.894	0.973	0.953
	LASSO	0.965	0.958	0.968	0.922	0.948	0.988	0.982
	Control	0.803	0.812	0.798	0.587	0.736	0.844	0.782

### 3.3 Analysis of the contribution of our multi-view features

**Fig 3:**
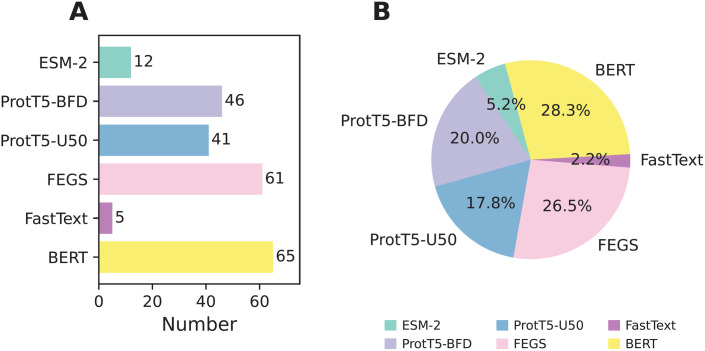
Analysis of the optimal feature set. The number (**A**) and of proportion (**B**) of each type feature embedding selected from the optimal feature set.

**Fig 4:**
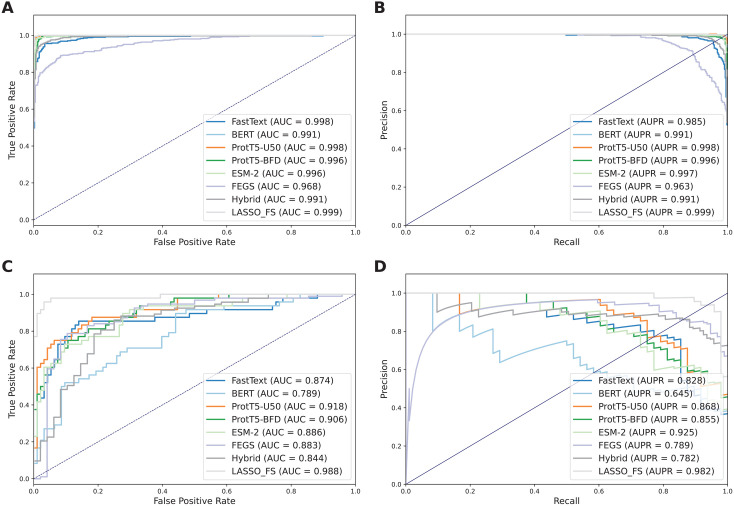
Performance comparison of different representations. Comparisons of the ROC curve, AUC value, PR curve, and AUPR value on the training (**A, B**) and independent test (**C, D**) datasets.

As mentioned above, our proposed feature subset (LASSO_FS), which combines multi-view information, is a 230-D feature vector derived from 5 FastText-based, 65 BERT-based, 41 ProtT5-U50-based, 46 ProtT5-BFD-based, 12 ESM-2-based, 61 FEGS-based features (as shown in Fig 3). To investigate the effectiveness of LASSO_FS, we compared its performance with six baseline feature descriptors. The performance results for LASSO_FS and the compared feature descriptors in both cross-validation and independent tests are summarized in Fig 4 and Table 3. As observed in Table 3, LASSO_FS achieved the best overall predictive performance across both cross-validation and independent tests. Compared to the best-performing baseline feature descriptor (ProtT5-U50) in the independent test, LASSO_FS achieved ACC, MCC, F1, AUC, and AUPR values of 0.965, 0.922, 0.948, 0.988, and 0.982, respectively, representing improvements of 10.56, 24.17, 16.58, 7.00, and 11.42%.

**Table 3:** Performance of different feature representations over the cross-validation and independent tests.

Evaluation strategy	Method	ACC	SN	SP	MCC	F1	AUC	AUPR
Cross-validation	FastText	0.951	0.968	0.934	0.904	0.952	0.988	0.985
	BERT	0.982	0.983	0.980	0.963	0.982	0.991	0.991
	ProtT5-U50	0.990	0.985	0.995	0.981	0.990	0.998	0.998
	ProtT5-BFD	0.982	0.985	0.978	0.964	0.982	0.996	0.996
	ESM-2	0.984	0.985	0.983	0.968	0.984	0.996	0.997
	FEGS	0.854	0.995	0.714	0.743	0.879	0.968	0.963
	Hybrid	0.947	0.990	0.905	0.900	0.951	0.991	0.991
	LASSO_FS	0.995	0.995	0.995	0.990	0.995	0.999	0.999
Independent test	FastText	0.852	0.771	0.894	0.668	0.779	0.874	0.828
	BERT	0.852	0.771	0.894	0.668	0.779	0.874	0.827
	ProtT5-U50	0.859	0.750	0.915	0.680	0.783	0.918	0.868
	ProtT5-BFD	0.845	0.750	0.894	0.651	0.766	0.906	0.855
	ESM-2	0.845	0.729	0.904	0.648	0.761	0.886	0.925
	FEGS	0.761	0.917	0.681	0.566	0.721	0.883	0.789
	Hybrid	0.803	0.812	0.798	0.587	0.736	0.844	0.782
	LASSO_FS	0.965	0.958	0.968	0.922	0.948	0.988	0.982

**Fig 5:**
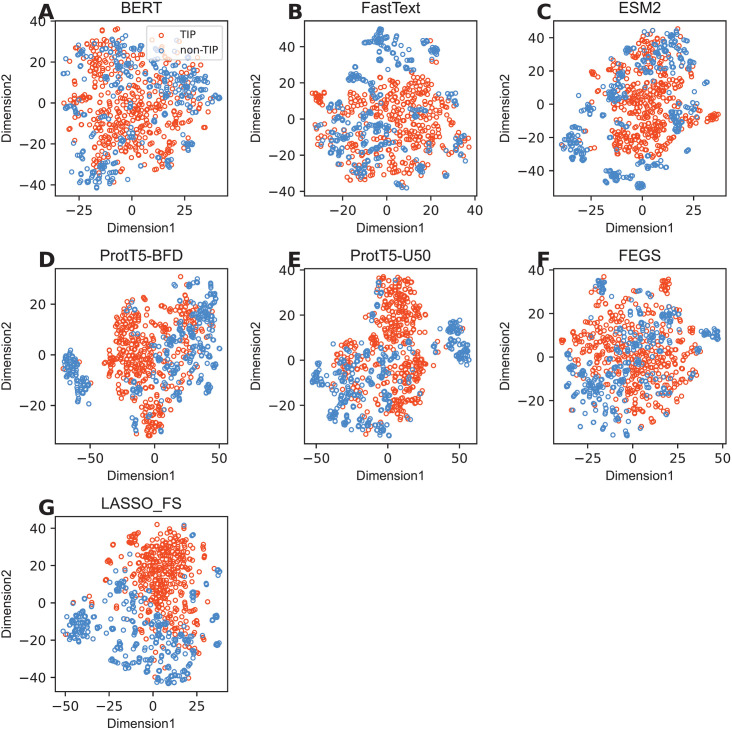
Comparison of t-SNE plots of our multi-view features (LASSO_FS) with other feature representations on the training dataset.

To further illustrate the effectiveness of LASSO_FS in distinguishing TIPs from non-TIPs, we visualized the distribution of TIPs and non-TIPs using the t-distributed stochastic neighbor embedding (t-SNE) method, which reduces the original feature space to a two-dimensional space [72]. Herein, seven t-SNE plots were created, as shown in Fig 5. It is evident that LASSO_FS (Fig 5G) forms two clear clusters of TIPs and non-TIPs, whereas unclear clusters were observed in the feature spaces of FastText, BERT, ProtT5-U50, ProtT5-BFD, ESM-2, and FEGS. Overall, these analysis results are sufficient to indicate that LASSO_FS effectively captures essential and sufficient information about TIPs. This explains why the proposed BLSAM-TIP model trained with LASSO_FS can precisely classify TIPs with great prediction performance.

### 3.4 Performance comparison between BiLSTM-TIP and several conventional ML and DL models

**Fig 6:**
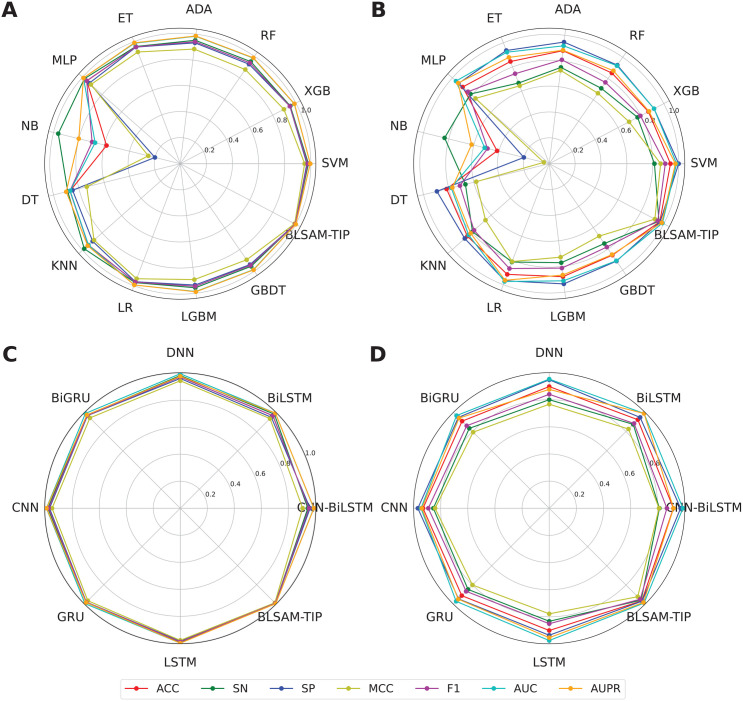
ACC, SN, SP, MCC, F1, AUC, AUPR scores of different prediction models on the training (A, C) and independent test (B, D) datasets. **(A-B)** Performance comparison of BLSAM-TIP with conventional ML models. **(C-D)** Performance comparison of BLSAM-TIP with conventional DL models.

To elucidate the effectiveness and robustness of the proposed BLSAM-TIP model, we compared its performance with several ML and DL classifiers using the same training and independent test datasets to ensure a fair comparison. In addition, all the compared ML and DL classifiers were constructed using the LASSO_FS feature subset and their optimal parameters, with the grid search space for each ML and DL classifier summarized in S2 and S3 Tables, respectively. Herein, we selected 12 ML methods (i.e., NB, DT, RF, KNN, ADA, LGBM, GBDT, XGB, MLP, ET, LR, and SVM) and 7 DL methods (i.e., CNN-BiLSTM, BiLSTM, DNN, BiGRU, CNN, GRU, and LSTM) to conduct the comparative experiments [18–20,73]. To date, these ML and DL methods have been widely and successfully applied to address numerous research questions in bioinformatics [20,38,48,49,74]. From Fig 6, Tables 4, 5, and S4 and S5 Tables, several key observations can be drawn as follows: (i) Among the top-five classifiers, almost all were based on DL methods (i.e., BiGRU, CNN, GRU, and LSTM), with the sole exception of SVM. The MCC values for BiGRU, CNN, SVM, GRU, and LSTM were 0.946, 0.949, 0.953, 0.968, and 0.978, respectively; (ii) On the independent test dataset, CNN and SVM still outperformed other classifiers, achieving MCC values of 0.841 and 0.861, respectively. The observation suggests that DL methods are particularly effective in leveraging information from large-scale datasets to attain impressive performance [38,74]; and (iii) When comparing BLSAM-TIP with CNN and SVM, BLSAM-TIP demonstrated slightly better performance. Specifically, BLSAM-TIP outperformed the compared models by 2.82–3.52% in ACC, 6.07–8.07% in MCC, 5.19–5.71% in F1, and 10.42–14.58% in SN. In summary, the proposed BLSAM-TIP model is more effective than several conventional ML and DL models in the identification of TIPs, especially in terms of performance on the independent test. These results indicate the excellent generalization ability and robustness of BLSAM-TIP.

**Table 4:** Comparison of the prediction results of BLSAM-TIP and conventional ML methods over the cross-validation and independent tests.

Method	Cross-validation test				Independent test			
	ACC	MCC	AUC	AUPR	ACC	MCC	AUC	AUPR
NB	0.582	0.252	0.672	0.801	0.415	0.043	0.514	0.618
DT	0.869	0.738	0.869	0.900	0.817	0.581	0.780	0.771
RF	0.939	0.877	0.984	0.986	0.852	0.662	0.921	0.873
KNN	0.940	0.883	0.940	0.948	0.845	0.658	0.832	0.811
ADA	0.942	0.885	0.984	0.986	0.880	0.727	0.918	0.884
LGBM	0.947	0.895	0.987	0.989	0.880	0.728	0.912	0.867
GBDT	0.947	0.895	0.987	0.989	0.859	0.680	0.912	0.854
XGB	0.949	0.897	0.987	0.988	0.866	0.697	0.915	0.871
MLP	0.957	0.915	0.991	0.991	0.894	0.761	0.963	0.940
ET	0.958	0.916	0.988	0.991	0.845	0.644	0.922	0.879
LR	0.972	0.943	0.994	0.994	0.915	0.809	0.974	0.964
SVM	0.977	0.953	0.996	0.996	0.937	0.861	0.984	0.975
BLSAM-TIP	0.995	0.990	0.999	0.999	0.965	0.922	0.988	0.982

**Table 5:** Comparison of the prediction results of BLSAM-TIP and conventional DL methods over the cross-validation and independent tests.

Method	Cross-validation test				Independent test			
	ACC	MCC	AUC	AUPR	ACC	MCC	AUC	AUPR
CNN-BiLSTM	0.952	0.906	0.988	0.989	0.915	0.809	0.985	0.916
BiLSTM	0.969	0.939	0.996	0.996	0.923	0.826	0.988	0.988
DNN	0.972	0.944	0.996	0.977	0.897	0.767	0.952	0.876
BiGRU	0.973	0.946	0.993	0.978	0.908	0.793	0.966	0.940
CNN	0.974	0.949	0.991	0.990	0.930	0.841	0.945	0.939
GRU	0.984	0.968	0.998	0.987	0.911	0.800	0.970	0.948
LSTM	0.989	0.978	0.999	0.999	0.901	0.778	0.975	0.954
BLSAM-TIP	0.995	0.990	0.999	0.999	0.965	0.922	0.988	0.982

### 3.5 Performance comparison between BLSAM-TIP and the existing methods

**Fig 7:**
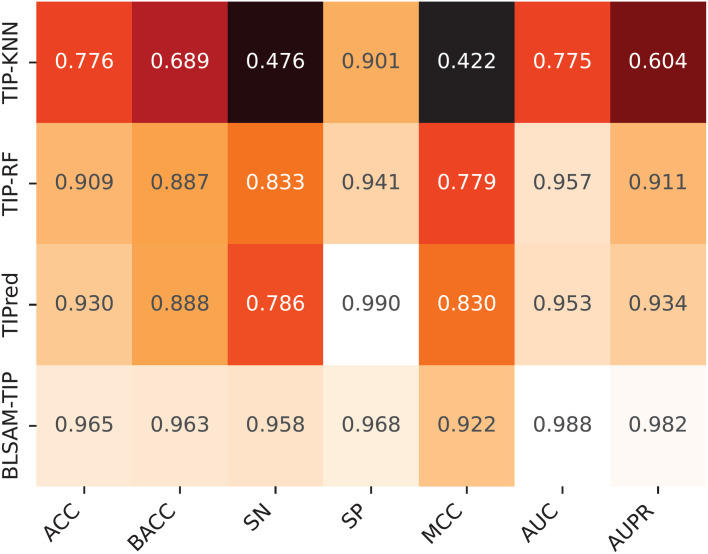
Heat-map of the prediction performance of our proposed BLSAM-TIP model and the existing methods over the independent test.

To reveal the excellent performance of the proposed BLSAM-TIP model, we compared it with existing methods, including TIP-KNN [15], TIP-RF [15], and TIPred [8], using the independent test, as summarized in Fig 7. Since TIP-KNN and TIP-RF are not available as online web servers for TIP identification, we implemented KNN-based and RF-based classifiers in conjunction with the selected feature encodings (i.e., AAC, DPC, and PCP). For TIPred, we evaluated its web server using the default threshold. As can be seen from Fig 7, BLSAM-TIP significantly outperformed all existing methods across nearly all performance metrics, including ACC, BACC, SN, MCC, AUC, and AUPR. To be specific, compared to the runner-up TIPred, BLSAM-TIP attained improvements of 7.53, 17.26, 9.18, 3.46, and 4.83% in BACC, SN, MCC, AUC, and AUPR values, respectively. Interestingly, the outstanding SN value of BLSAM-TIP underscores its ability to effectively minimize false negatives. Taken together, these results confirm that BLSAM-TIP delivers more stable and superior performance than the existing methods.

### 3.6 Ablation study

**Fig 8:**
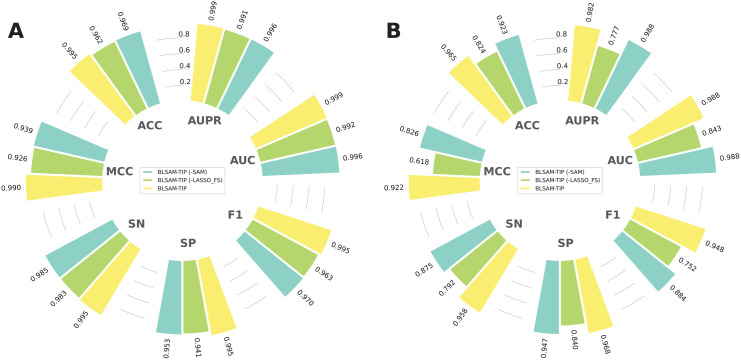
Comparison of the prediction results of BLSAM-TIP and its modified versions on the training (A) and independent test (B) datasets.

In this section, we performed ablation experiments to assess the contribution of our proposed computational approach for the accurate identification of TIPs. Specifically, we compared BLSAM-TIP with two modified versions: (i) BLSAM-TIP (-SAM) is the version of BLSAM-TIP trained without the use of SAM and (ii) BLSAM-TIP (-LASSO_FS) is the version of BLSAM-TIP trained using the original hybrid feature vector containing 3848 features instead of the optimized LASSO_FS subset. It can be noticed from Fig 8, BLSAM-TIP outperformed its modified versions in terms of ACC, SN, SP, MCC, and F1 scores in both cross-validation and independent tests. While BLSAM-TIP achieved comparable AUC and AUPR values to BSLAM-TIP (-SAM) in the cross-validation test, it demonstrated superior performance in the independent test. Specifically, BLSAM-TIP achieved ACC, SN, MCC, and F1 scores that were 4.23, 8.33, 9.57, and 6.42% higher than those of BLSAM-TIP (-SAM), respectively. These results confirm that the proposed computational approach benefits from the inclusion of individual components, such as SAM and LASSO_FS feature subset, enabling it to attain more accurate and robust TIP identification.

### 3.7 Case study

As can be seen in the above experiments, BLSAM-TIP consistently achieved stable and superior performance in TIP identification. In this section, we conducted case studies to investigate how effectively BLSAM-TIP can identify novel TIPs in unknown samples. Initially, we collected 11 experimentally validated TIPs from previous studies [75–82], while 67 new non-TIPs were peptides that had been experimentally validated as low or non-active, ranging from 5 to 20 amino acids in length [12,83] (S6 Table). Notably, these new peptides were not included in the training or independent test datasets, ensuring an unbiased assessment of our model’s generalization ability. The detailed prediction results of BLSAM-TIP and the existing methods (i.e., TIP-KNN, TIP-RF, and TIPred) in terms of the case studies are recorded in S7 Table. As can be seen from S7 Table, BLSAM-TIP outperformed all the existing methods. Specially, when compared with TIP-KNN and TIP-RF, BLSAM-TIP (ACC of 0.833) significantly outperformed these compared methods (ACC ranging from 0.295–0.654). This capability is important for prioritizing and ranking novel TIPs among large sets of uncharacterized peptides. Altogether, BLSAM-TIP shows clear superiority over the compared methods and holds promise as a computational tool for TIP identification.

## 4. Conclusions

This study presents BLSAM-TIP, a novel computational approach for the accurate and large-scale identification of TIPs by combining BiLSTM with SAM. Both cross-validation and independent tests confirm that BLSAM-TIP is an accurate and robust computational tool. In terms of the independent test, BLSAM-TIP significantly outperformed state-of-the-art methods for TIP identification, achieving a BACC of 0.936, MCC of 0.870, and AUC of 0.988. The excellent performance of BLSAM-TIP can be attributed to four major reasons: (i) Several feature encoding schemes from several perspectives are employed to capture multi-view and sufficient information about TIPs, including sequential, graphical, statistical, contextual, and protein semantic information; (ii) The SMOTE method is applied to handle the issue of class imbalance effectively; (iii) The LASSO-based feature subset contains excellent discriminative information, which contributes to significant performance improvements; and (iv) The BiLSTM-SAM-DNN architecture can effectively leverage the strengths of individual components to attain more accurate and stable TIP identification. Although BLSAM-TIP has greatly enhanced and facilitated TIP identification, there is still ample room for further improvement. One possible extension is to incorporate interpretable feature representations (such as physicochemical properties (PCPs) or amino acid and dipeptide propensities) into the current feature subset. Another potential enhancement is to implement the BLSAM-TIP web server to facilitate the *in-silico* identification of peptides with tyrosinase inhibitory properties.

## Supporting information

S1 Table A number of TIPs and non-TIPs used for developing three TIP predictors. (DOCX)

S2 Table Information of parameter settings for 12 ML methods used in this study. (DOCX)

S3 Table Information of parameter settings for five DL methods used in this study. (DOCX)

S4 Table Comparison of the prediction results of BLSAM-TIP and conventional ML methods over the cross-validation and independent tests. (DOCX)

S5 Table Comparison of the prediction results of BLSAM-TIP and conventional DL methods over the cross-validation and independent tests. (DOCX)

S6 Table Detailed information of new TIPs and non-TIPs in the case studies. (DOCX)

S7 Table Detailed prediction results of TIP-KNN, TIP-RF, TIPred, and BLSAM-TIP on case studies. (DOCX)
