# Supplementary material for: BLSAM-TIP: Improved and robust identification of tyrosinase inhibitory peptides by integrating bidirectional LSTM with self-attention mechanism
Source: PLoS One. 2025 Oct 8;20(10):e0333614. doi: 10.1371/journal.pone.0333614 (PMC12507286; doi:10.1371/journal.pone.0333614)
Supplement: S1 Table — (DOCX) [file pone.0333614.s001.docx]

## S1 Table A number of TIPs and non-TIPs used for developing three TIP predictors.

| **Class** | **Kongsompong et al** [1] | **Charoenkwan et al** [2] | **This study** |
| --- | --- | --- | --- |
| TIP | 133 | 133 | 206 |
| non-TIP | 13 | 287 | 502 |

### **Performance evaluation**

In this study, we assessed the predictive capabilities of several models using two established evaluation techniques: 10-fold cross-validation and independent test procedures. Therefore, we employed BACC, MCC, ACC, SN, SP, AUC, AUPR, and F1 [3-8]. These performance measures are defined as:

| $SN=\frac{\mathrm{TP}}{\left( TP+FN \right)}$ | (1) |
| --- | --- |
| $SP=\frac{\mathrm{TN}}{\left( TN+FP \right)}$ | (2) |
| $BACC=\frac{SN+SP}{2}$ | (3) |
| $MCC=\frac{TP\times TN-FP\times FN}{\sqrt{(TP+FP)(TP+FN)(TN+FP)(TN+FN)}}$ | (4) |
| $F1=2\times\frac{TP}{2TP+FP+FN}$ | (5) |
| $ACC=\frac{TP+TN}{\left( TP+TN+FP+FN \right)}$ | (6) |

where the numbers of correctly predicted TIPs and non-TIPs were referred as TP and TN, respectively. On the other hand, the numbers of falsely predicted TIPs and non-TIPs are referred to as FP and FN, respectively [9-11].

**References**

[1] S. Kongsompong, T. E-kobon, and P. Chumnanpuen, "K-Nearest Neighbor and Random Forest-Based Prediction of Putative Tyrosinase Inhibitory Peptides of Abalone Haliotis diversicolor," *Molecules,* vol. 26, no. 12, p. 3671, 2021.

[2] P. Charoenkwan, S. Kongsompong, N. Schaduangrat, P. Chumnanpuen, and W. Shoombuatong, "TIPred: a novel stacked ensemble approach for the accelerated discovery of tyrosinase inhibitory peptides," *BMC bioinformatics,* vol. 24, no. 1, p. 356, 2023.

[3] M. Azadpour, C. M. McKay, and R. L. Smith, "Estimating confidence intervals for information transfer analysis of confusion matrices," *The Journal of the Acoustical Society of America,* vol. 135, no. 3, pp. EL140-EL146, 2014.

[4] J. N. Mandrekar, "Receiver operating characteristic curve in diagnostic test assessment," *Journal of Thoracic Oncology,* vol. 5, no. 9, pp. 1315-1316, 2010.

[5] M. Ullah, K. Han, F. Hadi, J. Xu, J. Song, and D.-J. Yu, "PScL-HDeep: image-based prediction of protein subcellular location in human tissue using ensemble learning of handcrafted and deep learned features with two-layer feature selection," *Briefings in Bioinformatics,* vol. 22, no. 6, p. bbab278, 2021.

[6] F. Ge *et al.*, "MMPatho: Leveraging Multilevel Consensus and Evolutionary Information for Enhanced Missense Mutation Pathogenic Prediction," *Journal of Chemical Information and Modeling,* vol. 63, no. 22, pp. 7239-7257, 2023.

[7] M. Arif, G. Fang, A. Ghulam, S. Musleh, and T. Alam, "DPI_CDF: druggable protein identifier using cascade deep forest," *BMC bioinformatics,* vol. 25, no. 1, p. 145, 2024.

[8] P. Charoenkwan, N. Schaduangrat, P. Lio, M. A. Moni, P. Chumnanpuen, and W. Shoombuatong, "iAMAP-SCM: a novel computational tool for large-scale identification of antimalarial peptides using estimated propensity scores of dipeptides," *ACS omega,* vol. 7, no. 45, pp. 41082-41095, 2022.

[9] F.-Y. Dao, H. Lv, D. Zhang, Z.-M. Zhang, L. Liu, and H. Lin, "DeepYY1: a deep learning approach to identify YY1-mediated chromatin loops," *Briefings in bioinformatics,* vol. 22, no. 4, p. bbaa356, 2021.

[10] F.-Y. Dao *et al.*, "Identify origin of replication in Saccharomyces cerevisiae using two-step feature selection technique," *Bioinformatics,* vol. 35, no. 12, pp. 2075-2083, 2019.

[11] W. Chen, H. Lv, F. Nie, and H. Lin, "i6mA-Pred: identifying DNA N6-methyladenine sites in the rice genome," *Bioinformatics,* vol. 35, no. 16, pp. 2796-2800, 2019.
