# Supplementary material for: BLSAM-TIP: Improved and robust identification of tyrosinase inhibitory peptides by integrating bidirectional LSTM with self-attention mechanism
Source: PLoS One. 2025 Oct 8;20(10):e0333614. doi: 10.1371/journal.pone.0333614 (PMC12507286; doi:10.1371/journal.pone.0333614)
Supplement: S2 Table — (DOCX) [file pone.0333614.s002.docx]

## S2 Table Information of parameter settings for 12 ML methods used in this study.

| **Method** | **Parameter** | **Search space** |
| --- | --- | --- |
| ADA | n_estimators | [20, 50, 100, 200, 500] |
| DT | max_depth | 2–20 with an interval of 1. |
| ET | n_estimators | [20, 50, 100, 200, 500] |
| KNN | number of neighbours | 1–150 with an interval of 1 |
| LGBM | n_estimators | [20, 50, 100, 200, 500] |
| LR | C | np.logspace(-3, 3, num=100) |
| MLP | hidden_layer_sizes | [20, 50, 100, 200, 500] |
| NB | var_smoothing | np.logspace(0,-9, num=100) |
| PLS | #Components | 10–1000 with an interval of 10 |
| RF | n_estimators | [20, 50, 100, 200, 500] |
| SVM | Cost | [2^-4^–2^4^] in log_2_ steps |
| XGB | n_estimators | [20, 50, 100, 200, 500] |

Columns 2 and 3 represents the parameter name used in the Scikit-learn library and the range of parameter used to develop the model, respectively.
