# Supplementary material for: BLSAM-TIP: Improved and robust identification of tyrosinase inhibitory peptides by integrating bidirectional LSTM with self-attention mechanism
Source: PLoS One. 2025 Oct 8;20(10):e0333614. doi: 10.1371/journal.pone.0333614 (PMC12507286; doi:10.1371/journal.pone.0333614)
Supplement: S3 Table — (DOCX) [file pone.0333614.s003.docx]

## S3 Table Information of parameter settings for five DL methods used in this study.

| **Method** | **Parameter** | **Search Space** |
| --- | --- | --- |
| DNN | hidden_layers | [1, 2, 3, 4] |
|  | units_per_layer | [16, 32, 64, 128, 256, 512] |
|  | activation | ['relu','sigmoid', 'tanh'] |
|  | dropout_rate | [0.2, 0.3, 0.4, 0.5] |
| LSTM | units | [16, 32, 64, 128] |
|  | dropout_rate | [0.2, 0.3, 0.4, 0.5] |
|  | activation | ['relu',adam, adamx 'sigmoid', 'tanh'] |
| GRU | units | [16, 32, 64, 128] |
|  | dropout_rate | [0.2, 0.3, 0.4, 0.5] |
|  | activation | ['relu', 'sigmoid', 'tanh'] |
|  | optimizer | ['adam',’adam', ‘adamx’ , 'sgd', 'rmsprop'] |
| CNN | Filters (Conv1D) | [16, 32, 64, 128, 256] |
|  | Kernel Size | [3] |
|  | Activation | ['relu', 'sigmoid', 'tanh'] |
|  | Optimizer | ['adam', 'sgd', 'rmsprop', 'adamax'] |
|  | Learning Rate | [0.001, 0.01, 0.1] |
|  | Dropout Rate | [0.0, 0.2, 0.3, 0.5] |
| BiLSTM | Units | [32, 64, 128, 256] |
|  | Kernel Regularizer | [l2(0.01), l2(0.001), None] |
|  | Dropout Rate | [0.2, 0.5, 0.7] |
|  | Activation function | ['relu', 'sigmoid', 'tanh'] |
|  | Loss Function | ['binary_crossentropy', 'categorical_crossentropy'] |
|  | Optimizer | ['adam', 'sgd', 'rmsprop'] |
| CNN-BiLSTM | Filters | [16, 32, 64, 128, 256] |
|  | Kernel Size | [ 3] |
|  | Kernel Regularizer | [l2(0.01), l2(0.001), None] |
|  | Activation | ['relu', 'sigmoid', 'tanh'] |
|  | Batch Normalization | Applied after Conv1D |
|  | Dropout Rate | [0.2, 0.5, 0.7] |
|  | Units (GRU) | [8, 16, 32] |
|  | Dropout Rate (GRU) | [0.2, 0.5, 0.7] |
|  | Loss Function | ['binary_crossentropy','categorical_crossentropy'] |
|  | Optimizer | ['adam', 'sgd', 'rmsprop'] |

Columns 2 and 3 represents the parameter name used in the Scikit-learn library and the range of parameter used to develop the model, respectively.
