# Supplementary material for: BLSAM-TIP: Improved and robust identification of tyrosinase inhibitory peptides by integrating bidirectional LSTM with self-attention mechanism
Source: PLoS One. 2025 Oct 8;20(10):e0333614. doi: 10.1371/journal.pone.0333614 (PMC12507286; doi:10.1371/journal.pone.0333614)
Supplement: S4 Table — (DOCX) [file pone.0333614.s004.docx]

## S4 Table Comparison of the prediction results of BLSAM-TIP and conventional ML methods over the cross-validation and independent tests.

| **Evaluation strategy** | **Method** | **ACC** | **SN** | **SP** | **MCC** | **F1** | **AUC** | **AUPR** |
| --- | --- | --- | --- | --- | --- | --- | --- | --- |
| Cross-validation | NB | 0.582 | 0.963 | 0.200 | 0.252 | 0.696 | 0.672 | 0.801 |
|  | DT | 0.869 | 0.888 | 0.850 | 0.738 | 0.871 | 0.869 | 0.900 |
|  | RF | 0.939 | 0.951 | 0.927 | 0.877 | 0.939 | 0.984 | 0.986 |
|  | KNN | 0.940 | 0.983 | 0.897 | 0.883 | 0.942 | 0.940 | 0.948 |
|  | ADA | 0.942 | 0.953 | 0.933 | 0.885 | 0.942 | 0.984 | 0.986 |
|  | LGBM | 0.947 | 0.958 | 0.937 | 0.895 | 0.947 | 0.987 | 0.989 |
|  | GBDT | 0.947 | 0.956 | 0.940 | 0.895 | 0.947 | 0.987 | 0.989 |
|  | XGB | 0.949 | 0.952 | 0.948 | 0.897 | 0.948 | 0.987 | 0.988 |
|  | MLP | 0.957 | 0.982 | 0.931 | 0.915 | 0.958 | 0.991 | 0.991 |
|  | ET | 0.958 | 0.961 | 0.956 | 0.916 | 0.958 | 0.988 | 0.991 |
|  | LR | 0.972 | 0.975 | 0.968 | 0.943 | 0.971 | 0.994 | 0.994 |
|  | SVM | 0.977 | 0.982 | 0.970 | 0.953 | 0.976 | 0.996 | 0.996 |
|  | BLSAM-TIP | 0.995 | 0.995 | 0.995 | 0.990 | 0.995 | 0.999 | 0.999 |
| Independent test | NB | 0.415 | 0.833 | 0.202 | 0.043 | 0.491 | 0.514 | 0.618 |
|  | DT | 0.817 | 0.667 | 0.894 | 0.581 | 0.711 | 0.780 | 0.771 |
|  | RF | 0.852 | 0.708 | 0.926 | 0.662 | 0.764 | 0.921 | 0.873 |
|  | KNN | 0.845 | 0.792 | 0.872 | 0.658 | 0.776 | 0.832 | 0.811 |
|  | ADA | 0.880 | 0.750 | 0.947 | 0.727 | 0.809 | 0.918 | 0.884 |
|  | LGBM | 0.880 | 0.771 | 0.936 | 0.728 | 0.813 | 0.912 | 0.867 |
|  | GBDT | 0.859 | 0.750 | 0.915 | 0.680 | 0.783 | 0.912 | 0.854 |
|  | XGB | 0.866 | 0.771 | 0.915 | 0.697 | 0.796 | 0.915 | 0.871 |
|  | MLP | 0.894 | 0.813 | 0.936 | 0.761 | 0.839 | 0.963 | 0.940 |
|  | ET | 0.845 | 0.667 | 0.936 | 0.644 | 0.744 | 0.922 | 0.879 |
|  | LR | 0.915 | 0.813 | 0.968 | 0.809 | 0.867 | 0.974 | 0.964 |
|  | SVM | 0.937 | 0.813 | 1.000 | 0.861 | 0.897 | 0.984 | 0.975 |
|  | BLSAM-TIP | 0.965 | 0.958 | 0.968 | 0.922 | 0.948 | 0.988 | 0.982 |
