# Supplementary material for: BLSAM-TIP: Improved and robust identification of tyrosinase inhibitory peptides by integrating bidirectional LSTM with self-attention mechanism
Source: PLoS One. 2025 Oct 8;20(10):e0333614. doi: 10.1371/journal.pone.0333614 (PMC12507286; doi:10.1371/journal.pone.0333614)
Supplement: S5 Table — (DOCX) [file pone.0333614.s005.docx]

## S5 Table Comparison of the prediction results of BLSAM-TIP and conventional DL methods over the cross-validation and independent tests.

| **Evaluation strategy** | **Method** | **ACC** | **SN** | **SP** | **MCC** | **F1** | **AUC** | **AUPR** |
| --- | --- | --- | --- | --- | --- | --- | --- | --- |
| Cross-validation | CNN-BiLSTM | 0.952 | 0.944 | 0.961 | 0.906 | 0.952 | 0.988 | 0.989 |
|  | BiLSTM | 0.969 | 0.985 | 0.953 | 0.939 | 0.970 | 0.996 | 0.996 |
|  | DNN | 0.972 | 0.983 | 0.961 | 0.944 | 0.972 | 0.996 | 0.977 |
|  | BiGRU | 0.973 | 0.976 | 0.971 | 0.946 | 0.973 | 0.993 | 0.978 |
|  | CNN | 0.974 | 0.980 | 0.968 | 0.949 | 0.974 | 0.991 | 0.990 |
|  | GRU | 0.984 | 0.985 | 0.983 | 0.968 | 0.984 | 0.998 | 0.987 |
|  | LSTM | 0.989 | 0.993 | 0.985 | 0.978 | 0.989 | 0.999 | 0.999 |
|  | BLSAM-TIP | 0.995 | 0.995 | 0.995 | 0.990 | 0.995 | 0.999 | 0.999 |
| Independent test | CNN-BiLSTM | 0.915 | 0.812 | 0.968 | 0.809 | 0.867 | 0.985 | 0.916 |
|  | BiLSTM | 0.923 | 0.875 | 0.947 | 0.826 | 0.884 | 0.988 | 0.988 |
|  | DNN | 0.897 | 0.800 | 0.947 | 0.767 | 0.840 | 0.952 | 0.876 |
|  | BiGRU | 0.908 | 0.833 | 0.947 | 0.793 | 0.860 | 0.966 | 0.940 |
|  | CNN | 0.930 | 0.854 | 0.968 | 0.841 | 0.891 | 0.945 | 0.939 |
|  | GRU | 0.911 | 0.846 | 0.945 | 0.800 | 0.866 | 0.970 | 0.948 |
|  | LSTM | 0.901 | 0.833 | 0.936 | 0.778 | 0.851 | 0.975 | 0.954 |
|  | BLSAM-TIP | 0.965 | 0.958 | 0.968 | 0.922 | 0.948 | 0.988 | 0.982 |
