# Supplementary material for: BLSAM-TIP: Improved and robust identification of tyrosinase inhibitory peptides by integrating bidirectional LSTM with self-attention mechanism
Source: PLoS One. 2025 Oct 8;20(10):e0333614. doi: 10.1371/journal.pone.0333614 (PMC12507286; doi:10.1371/journal.pone.0333614)
Supplement: S6 Table — (DOCX) [file pone.0333614.s006.docx]

## S6 Table Detailed information of new TIPs and non-TIPs in the case studies

| **ID#** | **Class** | **Sequence** |
| --- | --- | --- |
| 1 | TIP | SFAPRFD |
| 2 | TIP | HYGR |
| 3 | TIP | SPGRLP |
| 4 | TIP | SSEYYGGEGSSSEQGYYGEG |
| 5 | TIP | CME |
| 6 | TIP | CRNL |
| 7 | TIP | AHYYD |
| 8 | TIP | DER |
| 9 | TIP | DEQERRRRRGRTRVRSE |
| 10 | TIP | DEQERRRRRGRTR |
| 11 | TIP | GLWGTLLGVAAGVAAIKL |
| 12 | non-TIP | DSDDKVS |
| 13 | non-TIP | ASTLSK |
| 14 | non-TIP | KVDDW |
| 15 | non-TIP | WNKAGE |
| 16 | non-TIP | NEEDIV |
| 17 | non-TIP | PDTEI |
| 18 | non-TIP | TLDQVAN |
| 19 | non-TIP | DETAA |
| 20 | non-TIP | WEDQI |
| 21 | non-TIP | FQAVI |
| 22 | non-TIP | QDDVG |
| 23 | non-TIP | DETAA |
| 24 | non-TIP | KITAA |
| 25 | non-TIP | QNSEK |
| 26 | non-TIP | GPSSN |
| 27 | non-TIP | PPKLVD |
| 28 | non-TIP | FIKGIE |
| 29 | non-TIP | WSRIASTWCIF |
| 30 | non-TIP | TFLQL |
| 31 | non-TIP | GPSSN |
| 32 | non-TIP | RDDKIQ |
| 33 | non-TIP | GTDIG |
| 34 | non-TIP | LPAYQ |
| 35 | non-TIP | KTIPI |
| 36 | non-TIP | EWIPW |
| 37 | non-TIP | NAWIS |
| 38 | non-TIP | KWYDH |
| 39 | non-TIP | SLLIW |
| 40 | non-TIP | QVIHMM |
| 41 | non-TIP | DMRFK |
| 42 | non-TIP | FWAPTA |
| 43 | non-TIP | ELPSGNGIIAA |
| 44 | non-TIP | VFHKKRGLM |
| 45 | non-TIP | EHCMP |
| 46 | non-TIP | QSIGIF |
| 47 | non-TIP | FQHDQLDMQCH |
| 48 | non-TIP | WVWNDGCILPL |
| 49 | non-TIP | ATPYG |
| 50 | non-TIP | QFWVGFPSWGD |
| 51 | non-TIP | MIQCDWW |
| 52 | non-TIP | NTRGQ |
| 53 | non-TIP | YEPATEVD |
| 54 | non-TIP | LAEWWMWGENV |
| 55 | non-TIP | QLAKLR |
| 56 | non-TIP | VEYSSFVENQD |
| 57 | non-TIP | EWQFDR |
| 58 | non-TIP | NQLWLMHEM |
| 59 | non-TIP | NLPNMNSDWGCC |
| 60 | non-TIP | FNQIWDVLAG |
| 61 | non-TIP | LPRDVHI |
| 62 | non-TIP | PAETQWSWGFMN |
| 63 | non-TIP | VNDIDFGCGPNA |
| 64 | non-TIP | IQWQNTGET |
| 65 | non-TIP | PILWGCGNSV |
| 66 | non-TIP | TTSTR |
| 67 | non-TIP | NNSSPPKGRT |
| 68 | non-TIP | NMESMF |
| 69 | non-TIP | PYPNAQESVDSVSEF |
| 70 | non-TIP | HLERRD |
| 71 | non-TIP | VAFPWGTRFR |
| 72 | non-TIP | DDYSLR |
| 73 | non-TIP | LWFTFGL |
| 74 | non-TIP | ICFGEPWCPIQ |
| 75 | non-TIP | TELTRMWDEGL |
| 76 | non-TIP | GDDKRPWPQWF |
| 77 | non-TIP | YEPQLQVLSAMC |
| 78 | non-TIP | CLRVEGVERYKE |
