# Supplementary material for: BLSAM-TIP: Improved and robust identification of tyrosinase inhibitory peptides by integrating bidirectional LSTM with self-attention mechanism
Source: PLoS One. 2025 Oct 8;20(10):e0333614. doi: 10.1371/journal.pone.0333614 (PMC12507286; doi:10.1371/journal.pone.0333614)
Supplement: S7 Table — (DOCX) [file pone.0333614.s007.docx]

## S7 Table Detailed prediction results of TIP-KNN, TIP-RF, TIPred, and BLSAM-TIP on case studies

| **#** | **True** | **TIP-KNN** | **TIP-RF** | **TIPred** | **BLSAM-TIP** |
| --- | --- | --- | --- | --- | --- |
| 1 | TIP | non-TIP | non-TIP | TIP | TIP |
| 2 | TIP | non-TIP | non-TIP | TIP | non-TIP |
| 3 | TIP | TIP | TIP | non-TIP | non-TIP |
| 4 | TIP | non-TIP | non-TIP | TIP | TIP |
| 5 | TIP | TIP | TIP | non-TIP | non-TIP |
| 6 | TIP | non-TIP | non-TIP | TIP | TIP |
| 7 | TIP | non-TIP | non-TIP | TIP | TIP |
| 8 | TIP | TIP | TIP | non-TIP | non-TIP |
| 9 | TIP | non-TIP | TIP | TIP | TIP |
| 10 | TIP | non-TIP | TIP | TIP | TIP |
| 11 | TIP | non-TIP | non-TIP | TIP | TIP |
| 12 | non-TIP | TIP | TIP | non-TIP | non-TIP |
| 13 | non-TIP | non-TIP | TIP | non-TIP | non-TIP |
| 14 | non-TIP | TIP | TIP | non-TIP | non-TIP |
| 15 | non-TIP | non-TIP | TIP | non-TIP | TIP |
| 16 | non-TIP | non-TIP | TIP | non-TIP | TIP |
| 17 | non-TIP | non-TIP | TIP | non-TIP | TIP |
| 18 | non-TIP | non-TIP | TIP | non-TIP | non-TIP |
| 19 | non-TIP | TIP | TIP | non-TIP | non-TIP |
| 20 | non-TIP | TIP | TIP | non-TIP | TIP |
| 21 | non-TIP | non-TIP | TIP | non-TIP | non-TIP |
| 22 | non-TIP | TIP | TIP | non-TIP | non-TIP |
| 23 | non-TIP | TIP | TIP | non-TIP | TIP |
| 24 | non-TIP | non-TIP | TIP | non-TIP | TIP |
| 25 | non-TIP | TIP | TIP | non-TIP | TIP |
| 26 | non-TIP | non-TIP | non-TIP | non-TIP | non-TIP |
| 27 | non-TIP | TIP | TIP | non-TIP | TIP |
| 28 | non-TIP | non-TIP | TIP | non-TIP | non-TIP |
| 29 | non-TIP | non-TIP | non-TIP | TIP | TIP |
| 30 | non-TIP | non-TIP | TIP | non-TIP | non-TIP |
| 31 | non-TIP | non-TIP | non-TIP | non-TIP | non-TIP |
| 32 | non-TIP | TIP | TIP | non-TIP | non-TIP |
| 33 | non-TIP | non-TIP | TIP | non-TIP | non-TIP |
| 34 | non-TIP | TIP | TIP | non-TIP | non-TIP |
| 35 | non-TIP | non-TIP | TIP | non-TIP | non-TIP |
| 36 | non-TIP | TIP | TIP | non-TIP | non-TIP |
| 37 | non-TIP | non-TIP | TIP | non-TIP | non-TIP |
| 38 | non-TIP | TIP | TIP | TIP | non-TIP |
| 39 | non-TIP | non-TIP | TIP | non-TIP | non-TIP |
| 40 | non-TIP | non-TIP | TIP | TIP | non-TIP |
| 41 | non-TIP | non-TIP | TIP | TIP | non-TIP |
| 42 | non-TIP | non-TIP | TIP | non-TIP | non-TIP |
| 43 | non-TIP | non-TIP | non-TIP | non-TIP | non-TIP |
| 44 | non-TIP | non-TIP | non-TIP | TIP | non-TIP |
| 45 | non-TIP | TIP | TIP | non-TIP | non-TIP |
| 46 | non-TIP | non-TIP | non-TIP | non-TIP | non-TIP |
| 47 | non-TIP | TIP | TIP | TIP | non-TIP |
| 48 | non-TIP | non-TIP | non-TIP | non-TIP | non-TIP |
| 49 | non-TIP | non-TIP | TIP | non-TIP | non-TIP |
| 50 | non-TIP | non-TIP | TIP | non-TIP | non-TIP |
| 51 | non-TIP | TIP | TIP | non-TIP | non-TIP |
| 52 | non-TIP | non-TIP | TIP | non-TIP | non-TIP |
| 53 | non-TIP | non-TIP | TIP | non-TIP | non-TIP |
| 54 | non-TIP | non-TIP | TIP | non-TIP | non-TIP |
| 55 | non-TIP | TIP | TIP | TIP | non-TIP |
| 56 | non-TIP | non-TIP | TIP | non-TIP | non-TIP |
| 57 | non-TIP | TIP | TIP | non-TIP | non-TIP |
| 58 | non-TIP | non-TIP | TIP | TIP | non-TIP |
| 59 | non-TIP | non-TIP | non-TIP | non-TIP | non-TIP |
| 60 | non-TIP | non-TIP | non-TIP | non-TIP | non-TIP |
| 61 | non-TIP | non-TIP | non-TIP | non-TIP | non-TIP |
| 62 | non-TIP | non-TIP | TIP | non-TIP | non-TIP |
| 63 | non-TIP | non-TIP | non-TIP | non-TIP | non-TIP |
| 64 | non-TIP | TIP | TIP | non-TIP | non-TIP |
| 65 | non-TIP | non-TIP | non-TIP | TIP | non-TIP |
| 66 | non-TIP | non-TIP | TIP | non-TIP | non-TIP |
| 67 | non-TIP | non-TIP | TIP | non-TIP | non-TIP |
| 68 | non-TIP | non-TIP | TIP | non-TIP | non-TIP |
| 69 | non-TIP | non-TIP | TIP | non-TIP | non-TIP |
| 70 | non-TIP | TIP | TIP | non-TIP | non-TIP |
| 71 | non-TIP | non-TIP | non-TIP | TIP | non-TIP |
| 72 | non-TIP | non-TIP | non-TIP | non-TIP | non-TIP |
| 73 | non-TIP | non-TIP | non-TIP | non-TIP | non-TIP |
| 74 | non-TIP | non-TIP | non-TIP | non-TIP | non-TIP |
| 75 | non-TIP | non-TIP | TIP | non-TIP | non-TIP |
| 76 | non-TIP | non-TIP | TIP | non-TIP | non-TIP |
| 77 | non-TIP | non-TIP | non-TIP | non-TIP | non-TIP |
| 78 | non-TIP | non-TIP | non-TIP | TIP | non-TIP |
